# 16. Pain in chronic pancreatitis

**DOI:** 10.1111/papr.70030

**Published:** 2025-04-06

**Authors:** Laura van Zeggeren, Raha Boelens Nabbi, Jan Willem Kallewaard, Monique Steegers, Steven P. Cohen, Leonardo Kapural, Hjalmar van Santvoort, André Wolff

**Affiliations:** ^1^ Department of Anesthesiology and Pain Medicine Rijnstate Hospital Arnhem The Netherlands; ^2^ Department of Anesthesiology, UMCG Pain Center, University Medical Center Groningen University of Groningen Groningen The Netherlands; ^3^ Department of Anesthesiology and Pain Medicine Rijnstate Hospital Elst The Netherlands; ^4^ Department of Anesthesiology and Pain Medicine Amsterdam University Medical Centers Amsterdam The Netherlands; ^5^ Department of Anesthesiology, Neurology, Physical Medicine & Rehabilitation, Psychiatry and Neurological Surgery Northwestern University Feinberg School of Medicine Chicago Illinois USA; ^6^ Department of Physical Medicine and Rehabilitation and Anesthesiology, Walter Reed National Military Medical Center Uniformed Services University of the Health Sciences Bethesda Maryland USA; ^7^ Carolinas Pain Institute Winston Salem North Carolina USA; ^8^ Department of Hepato‐Pancreato‐Biliary Surgery Regional Academic Cancer Center Utrecht Utrecht The Netherlands; ^9^ Department of Surgery St. Antonius Hospital Nieuwegein The Netherlands

**Keywords:** chronic pancreatitis, evidence‐based medicine

## Abstract

**Introduction:**

Chronic pancreatitis is defined as a disease of the pancreas in which recurrent inflammatory episodes result in replacement of the pancreatic parenchyma by fibrous connective tissue in individuals with genetic, environmental, and other risk factors. Pain is one of the most important symptoms of chronic pancreatitis and, in many cases, has chronic visceral nociceptive, nociplastic, and even neuropathic components, with evidence of both central and peripheral sensitization, neuroplasticity, and neurogenic inflammation.

**Methods:**

The literature on the diagnosis and treatment of pain in chronic pancreatitis was reviewed and summarized.

**Results:**

Treatment of abdominal pain in chronic pancreatitis is guided by pancreatic morphology on imaging, although the correlation between pain symptoms and pathoanatomical changes is not always straightforward. Patients with pancreatic duct obstruction are initially offered endoscopic or surgical therapies, while non‐obstructive disease is mostly managed medically. Lifestyle changes and psychological support are of particular importance for all chronic pancreatitis patients. Analgesic options range from non‐opioid medications to opioids and adjuvant agents. Interventional pain management may consist of radiofrequency treatment of the splanchnic nerves and spinal cord stimulation. To date, there are no randomized trials supporting their efficacy in the treatment of chronic pancreatitis pain, and the recommendation to consider these treatment options is justified by evidence from observational studies. Possible opioid‐sparing effects of interventional pain treatments are important to consider because opioid use and dependency are common in chronic pancreatitis patients and associated with worse outcomes. Celiac plexus block is not generally recommended for chronic pancreatitis due to the limited quality of evidence, overall short duration of effect, and invasiveness of the procedure. Central sensitization can impact the effectiveness of invasive treatments.

**Conclusions:**

Managing pain in chronic pancreatitis is a complex task that requires a multidimensional and individualized approach. Due to the lack of randomized trials, treatment decisions are often guided by expert opinion. Integrating pharmacological and non‐pharmacological interventions and collaborating with a multidisciplinary team are key components of effective chronic pancreatitis pain management.

## INTRODUCTION

The pancreas is a retroperitoneal organ located in the upper abdomen near the stomach, duodenum, aorta, vena cava, and left kidney. The pancreas has an endocrine function with production and secretion of insulin, glucagon, and somatostatin in the islets of Langerhans, and an exocrine function that involves the production and secretion of pancreatic digestive enzymes such as amylase, lipase, and trypsin in the acinar cells. Acini comprise units of approximately 20 acinar cells with a few scattered centroacinar cells. The centroacinar cells produce bicarbonate, which neutralizes gastric acid.

The regulation of the pancreas is controlled by hormones and the autonomic (i.e., sympathetic and parasympathetic) nervous system. The hormone cholecystokinin (CCK) is released from special intestinal cells and stimulates the exocrine function in the pancreas. The release of CCK is stimulated by CCK‐releasing peptide, a protein that is active at the intraluminal site and denatured by trypsin.

Innervation of the pancreas consists of nerves that arise from spinal afferent pancreatic neurons that enter the pancreas through sympathetic fibers from the splanchnic nerves and celiac plexus, as well as parasympathetic fibers from the vagus nerve. Parasympathetic fibers stimulate both exocrine and endocrine secretion. Sympathetic fibers have an inhibiting effect on this release. A rich sensory network is located in the pancreas around the acinar cells.^1^, ^2^, ^3^, ^4^

This narrative review is an update of the article “Pain in chronic pancreatitis” published in 2011.^3^ In 2016, an independent company, Kleijnen Systematic Reviews, performed a systematic review of the literature for the period 2010–2015, based on existing systematic reviews (SRs) and randomized controlled trials (RCTs). For the current article, an updated search was conducted for the period 2015–2024, using combinations of the following search terms: “chronic pancreatitis,” “abdomen/abdominal,” “pain,” “plexus coeliacus,” “celiac plexus,” “nervus splanchnicus,” “splanchnic nerve,” “spinal cord stimulation,” and various other treatment modalities. Additional, authors could select relevant missing articles from various sources.

### Chronic pancreatitis

Chronic pancreatitis is defined as a disease of the pancreas in which recurrent inflammatory episodes result in replacement of the pancreatic parenchyma by fibrous connective tissue in individuals with genetic, environmental, and other risk factors.^5^, ^6^, ^7^ This fibrotic reorganization causes progressive exocrine and endocrine pancreatic insufficiency and a variety of local and systemic complications, such as pseudocysts, pancreatic duct obstruction, and splanchnic venous thromboses, and may increase the risk for pancreatic cancer. Exocrine pancreatic insufficiency is characterized by steatorrhea and eventually weight loss, sarcopenia, and deficiencies of fat‐soluble vitamins and other micronutrients. Loss of islet mass and insulin leads to endocrine disturbances with glucose intolerance and diabetes. The chronic inflammatory state and deficiencies often result in osteopenia or osteoporosis.^8^, ^9^

In contrast to acute pancreatitis in which the acute inflammatory damage is transient, chronic pancreatitis is often a progressive condition. Although these two clinical pictures may overlap, they each have a different pathological picture, etiology, and clinical course.^3^ Approximately 3%–35% of patients with a first episode of acute pancreatitis will progress to chronic pancreatitis over the course of 3–8 years. Conversely, only about 50% of patients with chronic pancreatitis have previously documented episodes of acute pancreatitis, possibly because of a subclinical presentation.^10^

Chronic pancreatitis is not curable, and most patients will experience long‐standing symptoms. Almost 90% of chronic pancreatitis patients suffer from pain, which is associated with reduced quality of life, increased health resource utilization, and reduced life expectancy.^5^, ^6^, ^11^ Opioid dependence can be a particular problem in these patients, and is associated with polypharmacy, concurrent substance abuse, worse pain and treatment outcomes, and higher health care utilization.^12^, ^13^

### Common features of visceral pain

Visceral pain is often described as dull and diffuse and difficult to localize compared to somatic pain. This is because of the lack of distinct structure‐specific nociceptive pathways and the small somatotopic representation of visceral structures, leading to spinal cord convergence. Visceral pain is thus commonly associated with referred sensations and evokes strong autonomic and emotional responses, such as feelings of unpleasantness, anxiety, and fear.^14^, ^15^ Neural pathways that process sensory stimuli from visceral organs link autonomic input with emotional and cognitive centers in the brain, relying on integration from neuroendocrine and immunological inputs and sensory afferents.^16^ This bidirectional communication regulates visceral homeostasis and is described in terms of a “brain‐gut axis.”^17^

Unlike somatic pain, visceral organs lack specific pain receptors but possess organ‐specific low‐ and high‐threshold receptor systems, which are polymodal in nature and activated by mechanical (e.g., stretching, distension), chemical (e.g., acid, pH), and thermal stimuli. Whereas many primary afferent nerve fibers are initially unresponsive or minimally responsive to mechanical stimuli, they can become highly sensitive through various mechanisms of peripheral sensitization, such as in the presence of inflammation.^15^, ^18^, ^19^, ^20^

**FIGURE 1:**
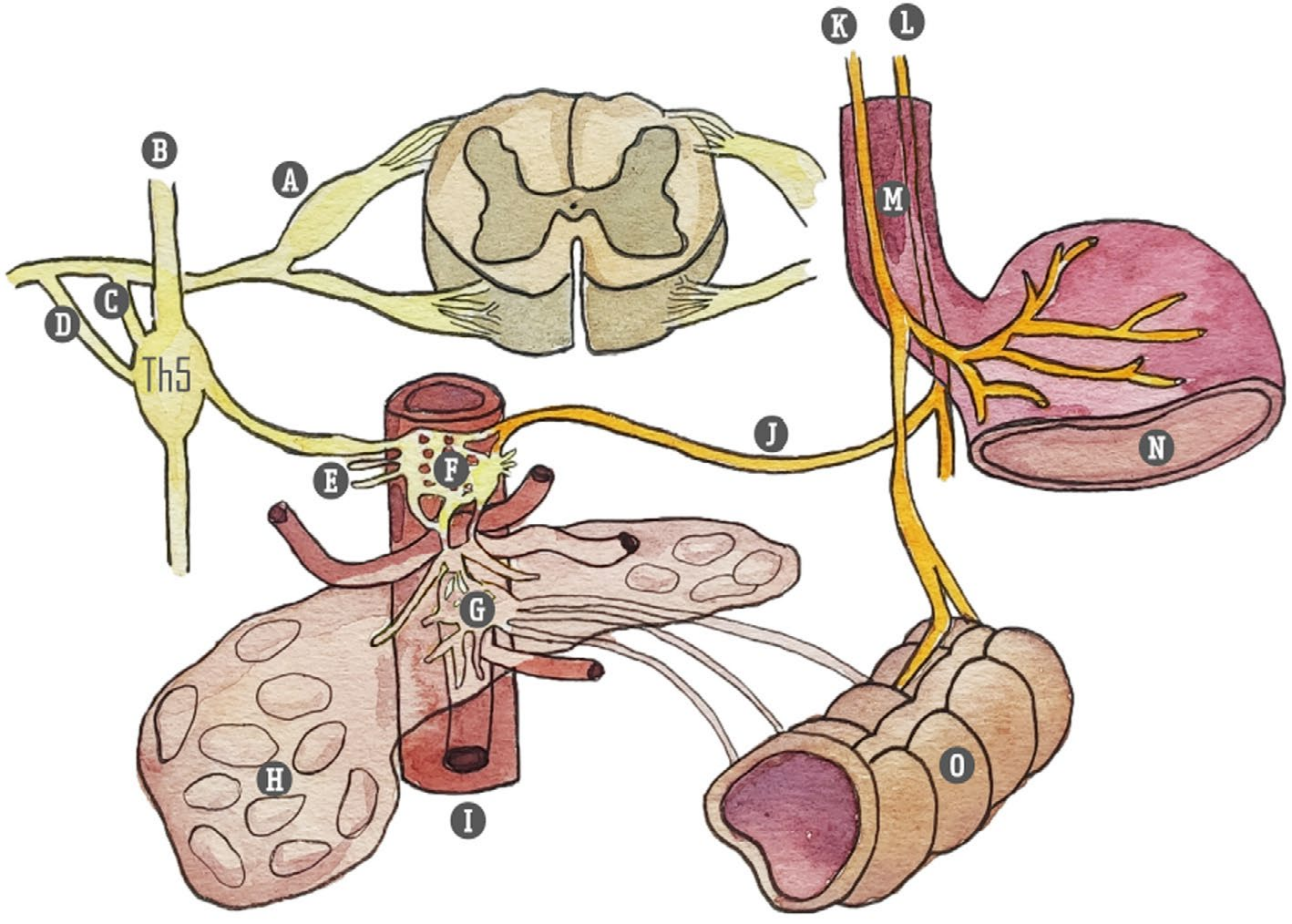
Innervation of pancreas. (A) Ganglion spinale (dorsal root ganglion); (B) truncus sympathicus; (C) white ramus communicantes; (D) gray ramus communicantes; (E) splanchnic nerves: Major (Th 5–9), minor (Th10–11), imus (Th‐12); (F) ganglion celiacum; (G) ganglion mesentericum superius; (H) pancreas; (I) aorta abdominalis; (J) branch to celiac plexus (truncus vagalis); (K) truncus vagalis anterior; (L) truncus vagalis posterior; (M) esophagus; (N) stomach; (O) colon. Illustration: Karien Brederode (adapted from Rogier Trompert, Medical Art, www.medical‐art.nl/).

Myelinated (Aδ) and unmyelinated (C) visceral sensory fibers travel from the viscera to the spinal cord via both parasympathetic pathways accompanying the vagus and pelvic nerves, and alongside sympathetic efferent fibers present in autonomic plexuses and the sympathetic trunk (Figure 1).^21^ The number of afferents projecting to visceral structures is relatively low when compared to non‐visceral somatic innervation, but visceral primary afferents have been demonstrated to branch extensively after they enter the spinal cord to reach multiple spinal segments above and below their point of entry. This leads to widespread and diffuse activation of the central nervous system. Second‐order processing occurs at spinal segments and brainstem sites that receive input from primary afferents. Visceral nociceptive information travels through both traditional spinothalamic pathways and through ipsilateral and dorsal spinal pathways. Relay sites for ascending information have been identified at multiple levels, including the medullary, pontine, mesencephalic, and thalamic levels. Furthermore, cortical processing of visceral information occurs in the insular cortex, anterior cingulate cortex (areas heavily involved in emotional processing) and secondary somatosensory cortex, among others.^14^, ^20^, ^22^ Conscious perception of signals from viscera, including pain, is present only when high‐threshold receptors are activated, typically by extreme distension.^15^

Sensitization in chronic visceral pain is intensified through several mechanisms. As alluded to above, *viscero‐somatic convergence* occurs when visceral afferent fibers merge with neurons located in the dorsal root ganglia (DRG), which contain cell bodies of sensory neurons from both visceral and somatic structures. Within the spinal cord, visceral and somatic sensory information also converge onto shared segments in lamina I and lamina V of the dorsal horn, where sensory neurons synapse with second‐order neurons. The input received from both visceral and somatic afferent fibers allows for integration and processing of sensory signals from different sources. Persistent nociceptive input from visceral organs may thus lead to sustained activation of somatic sensory pathways and vice versa, leading to abdominal wall sensitivity in individuals with chronic pancreatitis and similar pathology.^23^

*Viscero‐visceral convergence* refers to a similar concept wherein sensory information from different visceral organs projects onto shared neural pathways within the nervous system, at spinal as well as brainstem levels. Cross‐organ sensitization may occur as a result of this phenomenon.

The *convergence of sympathetic and sensory fibers* allows for communication between the sympathetic nervous system and sensory pathways originating from visceral organs. Sympathetic outflow may influence (e.g., amplify) the activity of sensory neurons and modulate the transmission of nociceptive signals, contributing to sensitization and plasticity of sensory pathways.^24^

*Dysfunctional autonomic regulation* such as sympathetic hyperactivity or parasympathetic dysfunction may affect normal patterns of motility and secretions, producing changes in the environment surrounding the nociceptive endings. Altered activity of viscera increases excitation of sensitized nociceptors and may even be sufficient to excite more distant nociceptors.

*Altered descending modulation and central pain processing* may further exacerbate sensitization and amplify viscero‐somatic interactions, contributing to the maintenance of a chronic pain state. Changes in brain structure and connectivity, especially in areas related to sensory processing and emotional regulation, are observed in functional gastrointestinal disorders, also suggesting a role in the pathophysiology of chronic visceral pain.^14^, ^15^, ^19^, ^20^

### Pain in chronic pancreatitis

The mechanisms of pain in chronic pancreatitis are complex, and although driven by pancreatic damage, they also involve changes in nociceptive function and central pain perception. Previously, it was assumed that increased pressure in pancreatic tissue or the ductal system could explain pain in most patients. However, no causal relationship has been shown between structural imaging findings and the intensity of pain.^3^, ^6^, ^25^ It is now recognized that the pain in chronic pancreatitis in many cases has a nociplastic component, with evidence of both central and peripheral neural sensitization, neuroplasticity, and neurogenic inflammation.^6^, ^8^, ^9^, ^26^, ^27^ In some patients, neuropathic pain mechanisms play a contributing role through structural, chemical, and inflammatory‐related injury to pancreatic nerves, along with ongoing nociceptive (including somatic pain) and nociplastic processes.^24^, ^28^

*Nociceptive pain* occurs when ongoing pancreatic inflammation causes the release of mast cells and inflammatory molecules, such as bradykinins, prostaglandins, and substance P, which then activate primary afferent neurons. Other factors that contribute to nociceptive pain in chronic pancreatitis include obstruction of the pancreatic duct due to stones or fibrosis, pseudocyst formation with compression or obstruction of the surrounding organs, and infiltration of the retroperitoneum.^3^, ^28^, ^29^ Pancreatic neurogenic inflammation – which can cause nociceptive pain (inflammation) and worsen nociplastic pain (sensitization) – can be caused by the activation of nerve endings and the secretion of inflammatory mediators, which decrease the pH, release inflammatory products such as ATP and cytokines, and are followed by perineural invasion and neuritis originating from damage to the perineurium. Neurogenic inflammation itself releases neuropeptides, which continue to reinforce inflammatory reactions in pancreatic tissue.^17^, ^24^, ^30^

*Neuropathic pain* involves injury or damage to the somatosensory system and may occur secondary to neural inflammation as noted above, or direct nerve injury (e.g., fibrosis and entrapment, ischemic injury with acute pancreatitis, chemical injury from altered acid–base status). Neuropathic pain can clinically be distinguished from nociceptive pain by descriptors (e.g., shooting, electrical‐like), regional allodynia or hyperalgesia, the presence of sensory deficits in the distribution of a nerve or nerve root, and quantitative sensory testing.

*Nociplastic pain* is pain that arises from altered nociception, which includes peripheral and central amplification of pain signals and decreased descending modulation.^26^ Nociplastic pain may involve functional and structural changes to the spinal cord and cortex, which can be both a consequence and a contributor to the pain burden.

The concept of pancreatic *neuroplasticity* describes the histopathological changes that are due to nerve stretching, resulting in a dense network of hypertrophic nerves and maladaptive changes in the dorsal root ganglia innervating the pancreas.^6^, ^24^

Acute or chronic pancreatitis can result in changes in the gut microbiome, and colonization of the pancreatic parenchyma.^31^ The relationship between pancreatic function and pathology, and gut microbiota is bidirectional and has been termed the “gut microbiota‐pancreas axis”. Changes in gut microbiota can predispose patients to, maintain, and magnify the entire spectrum of pain disorders including those involving nociceptive and inflammatory, neuropathic and nociplastic mechanisms, as well as influence the response to opioid and non‐opioid therapy.^32^, ^33^

Central sensitization is extremely common in chronic visceral pain syndromes and may manifest as reduced thresholds to stimulation of other viscera (e.g., gastrointestinal tract, bladder) and somatic structures such as muscles and bone.^6^ The use of validated instruments such as the Central Sensitization Inventory can be useful in the identification of a nociplastic phenotype.^34^

Peripheral and central sensitization can accelerate the development of chronic pain, resulting in visceral sensitivity, allodynia, and hyperalgesia.^8^, ^9^ Several studies found that electrical stimulation of the esophagus, stomach, duodenum, and colon in chronic pancreatitis patients results in amplified pain (hyperalgesia) and pain outside of typical referral patterns (expansion of receptive fields), and that chronic pancreatitis patients have reduced pain thresholds to somatic stimulation of muscle and bone.^35^, ^36^, ^37^, ^38^ The classical post‐prandial worsening of pain seen in many chronic pancreatitis patients may in fact represent allodynia triggered by the passage of food through the upper gut and stimulation of the pancreas.^39^

Taken together, these findings characterize a generalized hyperalgesic state and widespread sensitization of central sensory pathways. However, sequelae and complications such as pseudocysts, duodenal obstruction, bile duct obstruction, or a secondary pancreatic malignancy from chronic pancreatitis should not be overlooked.

### Epidemiology

According to the Dutch Pancreatitis Study Group, approximately 1000 patients are now diagnosed with chronic pancreatitis in the Netherlands each year.^40^ Internationally, the incidence varies from 4 to 8 new cases per 100,000 per year. The prevalence of chronic pancreatitis varies between studies and regions and is approximated at 15–125 people per 100,000 adults, with the highest reported prevalence in India.^7^, ^9^, ^10^, ^11^, ^41^ Since diagnosing chronic pancreatitis is challenging, these numbers may be underestimated because patients with abdominal complaints – without diagnostic classification or who are mistakenly diagnosed with other conditions such as irritable bowel syndrome – in reality may suffer from chronic pancreatitis.^6^, ^42^

Chronic pancreatitis is more common in men than in women and in Black individuals than in Caucasians, peaking at 46–55 years of age.^9^, ^43^

### Etiology

Although several causes of chronic pancreatitis have been described in the literature, most patients eventually develop typical clinical and morphological features.^10^ Risk factors include alcohol use (in 42%–77% of cases), smoking (over 60% in some studies), and genetic mutations (present in 10%). The etiology remains idiopathic in 28%–80% of cases, with genetic susceptibility and mutations often coexisting with environmental or behavioral risk factors in idiopathic cases.^7^, ^9^, ^10^, ^43^ Autoimmune pancreatitis and hereditary pancreatitis represent other (rare) causes of chronic pancreatitis.

While long‐standing alcohol consumption is a significant chronic pancreatitis risk factor, less than 5% of heavy drinkers develop the syndrome.^44^ Smoking is now recognized as an independent and equally impactful risk factor, and alcohol and smoking appear to act synergistically.^11^

Alcohol mediates its toxic effects directly on pancreatic acinar cells via its oxidative and non‐oxidative metabolites. In addition, ethanol induces microcirculatory disturbances and pancreatic ischemia, which mediate pancreatic acinar cell injury, activation of the inflammatory cascade, and a necro‐inflammatory response, resulting in fibrosis and chronic disease.^10^ Smoking contributes to acinar cell injury due to its nicotine‐derived, toxic metabolite nitrosamine ketone. Genetic mutations can cause cellular injury either in a trypsin‐dependent (loss or gain of function) or trypsin‐independent manner (e.g., bicarbonate secretion).^7^

A widely accepted theory, the so‐called *necrosis‐fibrosis theory*, suggests that an acute event triggers significant acinar cell stress or injury, resulting in clinically evident acute pancreatitis. Repeated pancreatic parenchymal injury and chronic inflammation contribute to fibrosis. Involvement of the pancreatic ducts leads to focal ductal strictures with proximal dilation, along with the formation of calculi due to secretory stasis and protein plug calcification. Ductal obstruction and recurrent injury cause parenchymal loss and further damage. This can also manifest as asymptomatic subclinical parenchymal injury and inflammation, with features of advanced disease secondary to repeated insults observed at initial presentation.

The *obstructive hypothesis* proposes that hypersecretion and protein precipitation cause protein plug formation in the pancreatic ducts, resulting in calcification and obstruction, which leads to acinar cell dysfunction and atrophy. Although sphincter of Oddi dysfunction was once considered a cause of pancreatitis, high recurrence rates and limited pain relief after invasive treatments have led to a decline in the use of these treatments.^7^

The natural history of chronic pancreatitis is not fully understood but is under investigation through ongoing prospective cohort studies, such as the PROCEED trial in the United States.^9^, ^45^ Recently, a study exploring risk factors for painful chronic pancreatitis found that young age, prolonged disease duration, heavy smoking, alcohol consumption, low body mass index, pancreatic exocrine insufficiency, pancreatitis attacks, anxiety and depression were associated with development of pain in chronic pancreatitis.^46^

## DIAGNOSIS

Diagnosing early‐stage chronic pancreatitis can be challenging due to preserved pancreatic function and subtle abnormalities in laboratory and imaging tests, often leading to a delay in diagnosis. Most diagnostic criteria have been developed using cases of advanced classic chronic pancreatitis, which may not suit the gradual development and early disease identification needed in routine clinical practice. In later stages of chronic pancreatitis, imaging tests become more reliable to confirm the diagnosis.^10^

Clinical suspicion of chronic pancreatitis is typically based on chronic or recurring pancreatic‐type pain (epigastric pain radiating to the back), steatorrhea, or diabetes. No definitive single diagnostic test for chronic pancreatitis exists; rather, it should be regarded as a syndrome. Imaging methods like computed tomography (CT), magnetic resonance cholangiopancreatography (MRCP), and endoscopic ultrasonography (EUS) are used to identify pancreatic changes characteristic of chronic pancreatitis and each has its specific advantages. Endoscopic retrograde cholangiopancreatography (ERCP) is no longer recommended for diagnostic purposes due to the risk of complications and the availability of noninvasive imaging options.^10^ CT, MRI, and EUS have similar sensitivity and specificity, but EUS is invasive, observer‐dependent, and more prone to false‐positive results.^10^, ^47^

Consensus guidelines suggest that if diagnostic imaging criteria are met, a diagnosis of chronic pancreatitis may be established by using imaging tests alone. In suspected cases with only probable imaging features, additional clinical criteria are required, including repeated upper abdominal pain, abnormal pancreatic enzyme levels, and evidence of pancreatic endocrine or exocrine dysfunction.^9^, ^10^

### History

In order to gain more insight into the underlying cause of chronic pancreatitis, a comprehensive clinical history is required, including details about alcohol and tobacco usage, as well as a personal and family history of pancreatitis or prior undiagnosed symptoms that might indicate a previous episode.^7^

Pain is the predominant symptom in chronic pancreatitis, affecting 75% of patients at presentation and nearly 100% over time, significantly impacting quality of life and functionality.^10^ Pain in chronic pancreatitis is variable and not always correlated with the extent of pathological or imaging changes.^48^ It can manifest differently in terms of severity, duration, and temporal patterns.^7^, ^49^, ^50^ Epigastric pain is present in the large majority of cases, with radiation to the back and hypochondrium also common.^51^ Early‐stage chronic pancreatitis is often marked by recurrent pain attacks associated with pancreatitis episodes and complications, followed by the development of chronic, debilitating pain in subsequent years. Advanced‐stage chronic pancreatitis is classically characterized by more persistent pain.^6^, ^9^

Patients can experience changing pain patterns over time, which may not be linked to medical or invasive interventions.^52^ Whereas some patients may achieve lasting pain relief, others continue to experience pain even after several years.^8^, ^41^

Severe pain in chronic pancreatitis can be debilitating and disrupt patients' lives, especially when compounded by alcohol use and psychosocial factors such as poor resilience and inadequate social support.^9^ Depression rates are high among chronic pancreatitis patients, and screening tools like the Hospital Anxiety and Depression Scale (HADS) can help identify cases.^53^

When managing pain in chronic pancreatitis, a multidimensional approach is important. History‐taking should not only include assessing pain intensity, pain patterns, and its impact on daily functioning and quality of life, but should also encompass substance abuse, resilience, motivation for behavioral change, and the disease's psychosocial effects.^9^, ^54^

### Physical examination

Generally, physical examination only reveals pain with pressure applied to the epigastrium. Fever or a palpable mass suggests a complicated course of chronic pancreatitis (pseudocyst).^3^

### Additional tests

#### Testing of pancreatic exocrine and endocrine function

Pancreatic exocrine and endocrine functions are important to address early in chronic pancreatitis management because of their association with malnutrition, diabetes mellitus, micronutrient depletion, and various symptoms such as diarrhea, bloating, and a risk for osteoporosis and cardiovascular events, which may affect quality of life.^10^

Lab tests such as amylase and lipase are often elevated (>3× normal levels) in acute pancreatitis, but in end‐state chronic pancreatitis, elevations may be blunted or even absent as a result of the destruction of pancreatic parenchyma. In terms of sensitivity, lipase has been shown to be superior to amylase for identification of acute pancreatitis and exacerbations of chronic pancreatitis. Although both are reasonably sensitive, both amylase and lipase are less accurate in chronic compared to acute pancreatitis and can also be elevated in other forms of visceral pain including mesenteric ischemia, biliary disease, and peptic ulcer exacerbations.^55^

#### Pain assessment

Assessing pain in chronic pancreatitis involves using multidimensional tools to capture the complexity of the pain. While the numerical pain rating scale (NRS) is commonly used, tools like the Brief Pain Inventory (BPI) or McGill Pain Questionnaire are preferred for a more extensive assessment.^10^ The Comprehensive Pain Assessment Tool (COMPAT) questionnaire has also been validated for chronic pancreatitis‐related pain.^8^

For assessing the impact of pain on quality of life, questionnaires like the European Organization for Research and Treatment of Cancer (EORTC) QLQ‐C30 and the Pancreatitis Quality‐of‐Life Instrument (PANQOLI) have been validated in individuals with chronic pancreatitis.^8^, ^56^, ^57^ The Izbicki pain score has not been officially validated in chronic pancreatitis, but is a helpful tool to quantify pain intensity, interference with daily activities, and emotional impact, providing insights into the complex nature of chronic pancreatitis pain.^41^ Since chronic pancreatitis patients often have other pain comorbidities and symptoms of central sensitization, the Central Sensitization Inventory can be used to identify patients with a nociplastic component and help guide treatment.^34^

In general, assessment of pain in chronic pancreatitis can be done in many ways and depends on the research questions asked in the specific trials or clinical settings. Recommendations have been put forward in an international consensus guideline.^58^

#### Pancreas specific quantitative sensory testing (P‐QST)

Quantitative sensory testing (QST) can be an additional tool for assessing pain and quantifying sensitivity. It has been used to identify patients with central sensitization in other painful conditions and can be useful in distinguishing neuropathic and nociplastic from non‐neuropathic pain.^48^ Earlier experimental QST protocols in chronic pancreatitis patients identified central sensitization as a predictor of poorer interventional treatment outcomes^10^, ^48^, ^59^, ^60^ and predictive of treatment response to medications such as pregabalin.^35^, ^61^ A specific QST protocol for chronic pancreatitis, known as P‐QST, has recently been developed to evaluate the various components of visceral pain.^62^, ^63^ However, its role in treating chronic pancreatitis pain has yet to be elucidated. Application could be integrated with other clinical and psychosocial assessment tools to better assist in detecting the presence of central sensitization in the various subtypes.^8^, ^48^

#### Scoring severity and disease activity

Evaluating disease severity and activity in chronic pancreatitis is a clinical challenge due to the absence of a widely accepted clinical tool or classification system for predicting short‐ and intermediate‐term outcomes. The M‐ANNHEIM classification and the Chronic Pancreatitis Prognosis Score are the only classification systems that include a severity index.^8^, ^10^ Although several chronic pancreatitis classification schemes have been proposed, no consensus exists among international experts regarding the definition of early chronic pancreatitis. International guidelines suggest the need for prospective studies to address this issue.

### Differential diagnosis

Differential diagnosis of chronic pancreatitis includes functional gastrointestinal disorders such as irritable bowel syndrome, peptic ulcer, symptomatic gallstone disease, and anterior cutaneous entrapment syndrome. Acute and chronic pancreatitis are frequently co‐prevalent with inflammatory bowel disease, including but not limited to the autoimmune subtype; these may overlap in their presentation, and physicians should be aware of the association in individuals with Crohn's disease or ulcerative colitis.^64^ Patients with chronic pancreatitis also have a higher risk of pancreatic cancer, particularly those with certain genetic forms or those who engage in excessive drinking or smoking. Surveillance is not universally recommended, but symptom changes (weight loss, bleeding associated with abdominal pain) should prompt evaluation for secondary pancreatic cancer. Pancreatic and extra‐pancreatic complications (stones, strictures, inflammatory masses, pseudocysts, gastrointestinal cancer) can contribute to pain and require thorough investigation during diagnosis and if pain worsens.^54^ An overview of differential diagnoses of painful chronic pancreatitis is shown in Table 1.

**TABLE 1:** Differential diagnosis of painful chronic pancreatitis.

Functional gastrointestinal disorders (such as irritable bowel syndrome)
Peptic ulcer
Symptomatic gallstone disease
Anterior cutaneous entrapment syndrome (ACNES)
Inflammatory bowel disease
Crohn's disease
Ulcerative colitis
Mesenteric ischemia
Gastrointestinal cancer
Pancreatic cancer
Pancreatic and extra‐pancreatic complications
Stones, strictures, inflammatory masses, pseudocysts

### Treatment options

Treatment of abdominal pain is guided by pancreatic morphology on imaging. Patients with pancreatic duct obstruction are offered endoscopic or surgical therapies, while nonobstructive chronic pancreatitis is mostly managed medically. Analgesic options range from non‐opioid medications to opioids and adjuvant agents.

Due to the lack of randomized trials specifically targeting chronic pancreatitis pain, treatment decisions are often guided by expert opinion and adaptations from pain management strategies used for other conditions. Central sensitization can impact the efficacy of invasive treatments. Integrating pharmacological and non‐pharmacological interventions and collaborating with a multidisciplinary team are key components of effective chronic pancreatitis pain management.

#### Conservative management

##### Lifestyle adjustments

Implementing lifestyle adjustments is crucial for effectively managing chronic pancreatitis. It is highly recommended that patients cease alcohol consumption and quit smoking. Patients should be provided with counseling and support to refrain from these substances because alcohol and smoking abstinence not only slows down the progression of chronic pancreatitis but also lowers the risk of developing malignancies and generally decreases pain levels.^7^, ^8^, ^10^, ^11^, ^41^, ^48^ Adopting a low‐fat diet is often recommended, although randomized studies are lacking. Maintaining proper nutrition is essential to prevent muscle loss and vitamin deficiencies, which can have significant implications for chronic pancreatitis patients.^11^

##### Analgesics

Analgesics are often the first step in managing pain associated with chronic pancreatitis, although their efficacy has not always been conclusively proven through randomized trials. The WHO analgesic ladder, designed for cancer pain, is often applied due to the lack of specific guidelines.^54^ Various analgesic options are available, and treatment strategies depend on individual patient characteristics, the severity of symptoms, and suspected underlying mechanisms of pain. As chronic pancreatitis is classically visceral nociceptive pain, paracetamol, nonsteroidal anti‐inflammatory drugs (NSAIDs) or tramadol can be considered as an initial approach (step 1 and 2). If there is a substantial neuropathic contribution to pain or the presentation shows features of central sensitization, then anti‐neuropathic pain medications and N‐methyl‐D‐aspartate (NMDA) receptor antagonists may have opioid‐sparing, analgesic effects (step 4 and 5).

Analgesic options:

1. Non‐opioid analgesics: Paracetamol (acetaminophen) is commonly used as a first‐line analgesic due to its mild side effects. Nonsteroidal anti‐inflammatory drugs and selective COX‐2 inhibitors can also be considered, but their use is limited due to potential renal, cardiovascular, and gastrointestinal side effects, especially in patients already predisposed to peptic ulcers. Metamizole represents another potent non‐opioid analgesic that possesses spasmolytic effects and a more favorable gastrointestinal side effect profile, which can be beneficial for painful chronic pancreatitis. Concerns have been raised about agranulocytosis as a major adverse effect (and more recently drug‐induced liver injury^65^, ^66^), and therefore, the drug is not approved in many countries.^8^, ^10^
2. Mixed action opioids: Tramadol, a weak opioid with a ceiling effect that inhibits the reuptake of norepinephrine and serotonin, is frequently utilized as a second‐line treatment in chronic pancreatitis. It offers a balance between analgesic efficacy and side effects and is superior to morphine in terms of gastrointestinal tolerability.^8^, ^67^ Buprenorphine, a partial agonist at mu and antagonist at the kappa receptor that is available in patch and transmucosal formulations, might be considered as an alternative.^68^, ^69^ These properties are associated with a reduction in gastrointestinal effects compared to orally administered, pure mu agonist opioids.
3. Opioids such as morphine that are full agonists at the mu and other opioid receptors are used to manage severe pain in chronic pancreatitis. However, the potential for dependency, side effects including constipation, bloating, and nausea and vomiting which may already be present in chronic pancreatitis patients, and the risk of opioid‐induced hyperalgesia necessitate caution.^9^ It has been shown that up to half of chronic pancreatitis patients use opioids for pain and that these numbers may have increased in the last two decades.^12^, ^25^, ^70^ Opioid treatment in chronic pancreatitis patients is often complicated by altered metabolism due to compromised liver and gut function. The lowest possible dose should be used and notably, in up to 50% of chronic pain patients in general, opioids do not alleviate pain and treatment should be stopped.^54^
4. Gabapentinoids and antidepressants: Adjuvant analgesics such as gabapentinoids (e.g., gabapentin, pregabalin) and tricyclic antidepressants have shown effectiveness in managing nociplastic, neuropathic, and occasionally visceral pain in chronic pancreatitis.^10^, ^41^, ^59^ Of these, only pregabalin has been studied in a randomized controlled trial in patients with chronic pancreatitis. Among 64 enrolled patients, the pregabalin‐treated group exhibited a 36% pain reduction score at 3 weeks compared with a 24% pain reduction score in the placebo group.^7^, ^8^, ^38^
5. Ketamine or lidocaine: In some cases, treatments like lidocaine or ketamine infusions have been considered beneficial for pain relief, particularly under the guidance of pain specialists and when opioid analgesics are ineffective or producing opioid‐induced hyperalgesia. Ketamine's antagonistic effects on NMDA receptors can improve pain control, reduce opioid requirements, and possibly reinstate opioid effectiveness at lower dosing.^35^, ^71^, ^72^ Unfortunately, an attempt to investigate the effects of ketamine in chronic pancreatitis patients in a recent double‐blinded, placebo‐controlled randomized trial was ended prematurely due to a lack of enrollment.^73^ A recent multicenter, prospective pilot study investigated the effects of intravenous lidocaine in patients with pancreatic cancer (19 patients) and chronic pancreatitis pain (11 patients).^74^ Only 2 of 11 chronic pancreatitis patients (18%) were considered responders with a relevant decrease in BPI (≥1.3) on the first day after infusion. However, at 1 and 3 months after the treatment, clinically relevant mean differences in BPI and pain severity were observed in the chronic pancreatitis group. Only minor side effects were reported. The authors conclude that larger studies with greater sample sizes and less heterogeneity are needed to prove or disprove the efficacy of intravenous lidocaine in chronic pancreatitis patients.

##### Enzyme suppletion

The use of pancreatic enzyme supplementation is based on the hypothesis that they degrade CCK‐releasing factor, which in turn reduces CCK release and pancreatic secretion, thereby decreasing intraductal pressure in the setting of ductal obstruction.^41^ Whereas enzyme replacement therapy (ERT) can alleviate symptoms of maldigestion, its efficacy for pain relief is debatable. Randomized trials have yielded mixed results, with some studies showing pain reduction when using uncoated enzyme preparations but not with enteric‐coated formulations.^75^ Consequently, guidelines are mixed in their recommendations of ERT for pain relief in chronic pancreatitis.^41^, ^54^ However, there are indications in which enzyme supplementation may alleviate abdominal discomfort related to exocrine insufficiency, such as with excessive flatus and bloating.^8^, ^9^, ^10^, ^76^

##### Octreotide

Somatostatin‐analogues, such as octreotide, inhibit pancreatic secretion and may theoretically alleviate pain by reducing pancreatic ductal pressure. However, data on effectiveness are conflicting.^41^

##### Antioxidant therapy

Reactive oxidative species (ROS) are natural byproducts of aerobic metabolism. When production increases due to pathologic conditions, oxidative stress‐induced injury occurs. It is well known that oxidative stress plays a role in the pathogenesis of chronic pancreatitis.^41^

Meta‐analyses of randomized trials evaluating various commercially available antioxidant combinations (vitamin A, C, and E, and S‐adenosyl‐methionine) have shown significant decreases in the number of days with pain and a reduction in opioid consumption.^77^, ^78^ However, these trials included small numbers of patients, and one trial showed no benefit. The recommendation to use these is mostly based on the potential benefits in the absence of adverse effects.^9^ Combination therapy (rational polypharmacy) may provide additive effects, with one study finding that a combination of pregabalin and antioxidants resulted in lower pain scores, a higher proportion of complete pain responders, and less painful days in those who experienced a recurrence of pain after surgical or endoscopic therapy.^79^

##### Vagal nerve stimulation

Studies have demonstrated that transcutaneous vagal nerve stimulation (VNS) provides anti‐nociceptive, anti‐inflammatory, and antidepressant effects, which can be beneficial in patients with chronic pancreatitis. Although its mechanism has not been fully elucidated, VNS has already been explored as a potential treatment option in other chronic pain conditions that share a strong nociplastic component, including fibromyalgia, pelvic pain, and headache.^80^, ^81^, ^82^ Regarding chronic pancreatitis, short‐term VNS has been evaluated in one randomized controlled study, which found no significant difference in pain relief between VNS and sham treatment.^83^ Another exploratory study demonstrated no effect on pain sensitivity.^84^ In conclusion, the effect of VNS on chronic pancreatitis is at best inconclusive, with further studies warranted.

##### Acupuncture

The mechanisms of action underlying acupuncture analgesia are not well known. One theory proposes that activation of endogenous opioid systems may play a role and influence the state of cortical pain processing. Limited studies exist comparing real acupuncture to placebo acupuncture in the context of chronic pancreatitis. One recent prospective, randomized, single‐blind crossover trial conducted in 15 patients found “proof‐of‐concept” for the analgesic effect of acupuncture for chronic pancreatitis pain, although the effect was short‐lasting.^84^ Considering its safety and lack of side effects, acupuncture could be a promising option for well‐selected chronic pancreatitis patients.^54^

##### Psychological interventions

Social factors and psychological comorbidities play a significant role in the experience of pain in chronic pancreatitis. Anxiety, depression, and to a lesser extent, posttraumatic stress disorder are highly co‐prevalent among chronic pancreatitis patients and are associated with higher pain severity.^63^ Cognitive behavioral therapy (CBT) has been effective in managing chronic pain in various conditions, including chronic pancreatitis.^85^ Guidelines recommend incorporating psychosocial interventions as part of a multidisciplinary approach to chronic pancreatitis pain management.^41^ An internet‐based CBT pilot trial performed specifically in chronic pancreatitis patients reported a significant decrease in pain intensity and pain interference compared to a control group.^86^

#### Interventional management

##### Endoscopic and surgical treatment options

Endoscopic or surgical treatment for chronic pancreatitis pain is often considered when conservative treatment is inadequate or when morphological changes (obstruction, inflammatory mass head) are present. However, the correlation between pain symptoms and structural (imaging) changes is not always straightforward, and none of the available interventional trials – endoscopic nor surgical – included a sham intervention as a control, resulting in potential selection bias and probably a robust placebo effect. Optimal timing and whether surgical or endoscopic therapy should be offered first is a matter of ongoing debate. The choice should depend on patient characteristics and preference, disease severity, and local expertise.

###### Endoscopic therapy

Endoscopic interventions primarily involve removing pancreatic duct stones, dilating strictures, or both. Extracorporeal shockwave lithotripsy (ESWL) may be used to break up larger stones before endoscopic removal. Pain relief can be achieved in a substantial number of patients through these interventions.^87^, ^88^, ^89^ Endoscopic therapy is less invasive compared to surgery and is often preferred as the initial treatment option.^7^ It may also be used for complications like pseudocysts and pancreatic fluid collections.^90^, ^91^

###### Surgery

Surgical procedures for pain relief in chronic pancreatitis include various approaches, including partial resection, drainage procedures, and combined resection and drainage.^92^ The type of surgery chosen depends on factors such as the extent of inflammation, ductal obstruction, and local preferences. Surgical outcomes tend to be better when patients are referred within the first 3–5 years from the onset of symptoms and before opioid dependence develops.^52^, ^93^ Total pancreatectomy with islet autotransplantation (TPIAT) has gained attention over the past decade and involves removing the pancreas and infusing isolated islets back into the recipient's portal vein to mitigate diabetes. TPIAT has been associated with durable pain relief in between 63% and 90% of selected patients and is considered mainly for those with genetic or idiopathic disease and intractable pain.^52^, ^94^, ^95^

###### Comparing surgery and endoscopy

Two randomized trials of earlier date, involving patients with chronic pancreatitis and ductal obstruction showed that surgery offered greater pain relief compared to endoscopic interventions.^96^, ^97^ A Cochrane review, incorporating these trials that included 111 patients with follow‐up data, demonstrated that surgery had a higher pain relief rate at 2–5 years and beyond compared to endoscopic procedures.^98^ An explanation for this outcome might be that the surgical method not only alleviates ductal pressure by enabling drainage, but also removes inflamed tissue that induces neural alterations and pain.^10^ The other study in the Cochrane review found surgery to be superior to conservative management in 32 patients with painful obstructive chronic pancreatitis.^99^

In the more recent Dutch ESCAPE trial (88 patients randomized), early surgery within 2–6 months of commencing opioids was compared to a stepwise approach involving medical therapy and endoscopy.^100^ The findings showed better short‐term pain relief in the early surgery group.

##### Interventional pain management

Interventional pain management can provide pain relief when conventional analgesics are insufficient, cause side effects or when chronic pain persists after endoscopy or surgery.^101^, ^102^ Some authors have raised concerns about whether any local neuroablative procedure can provide lasting relief in the setting of a self‐perpetuating pain state with ongoing sensitization. These concerns include the possible development of ‘deafferentation pain’ (unlikely with a small pancreatic somatotopic representation in the cortex), neuroma formation and fibrosis, and rerouting of damaged nerve connections after (repeated) neurolytic treatments.^8^, ^103^, ^104^ In contrast, others advocate the use of invasive, interventional treatments earlier in the algorithm of pain management due to their ability to provide intermediate‐term pain relief with limited side effects and risks, and possibly preventing opioid use disorder and central sensitization.^29^

This review will provide an overview of the current evidence for the following interventional techniques for chronic pancreatitis pain:

1. Celiac plexus block
2. Splanchnic nerve block and radiofrequency treatment
3. Spinal cord stimulation

Nerve blocks may be performed with local anesthetics and/or corticosteroids (temporary or diagnostic/prognostic blocks), whereas neurolytic interventions consist of chemical neuroablative injections performed with alcohol, phenol, or glycerol, or thermal radiofrequency lesioning.

Studies using any comparators, including sham, no treatment, or other active treatment techniques, were eligible for consideration.

###### Relevant anatomy

**FIGURE 2:**
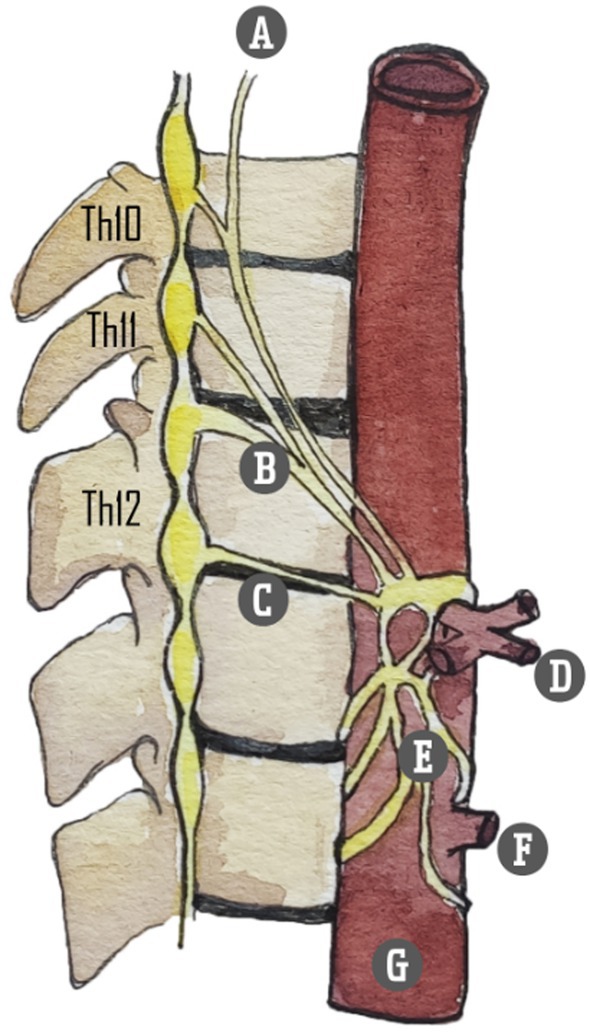
Splanchnic nerves. (A) Greater splanchnic nerve; (B) lesser splanchnic nerve; (C) least splanchnic nerve; (D) celiac trunc; (E) celiac plexus; (F) superior mesenteric artery; (G) aorta abdominalis. Illustration: Karien Brederode (adapted from Garcea G, Thomasset S, Berry DP, Tordoff S. Percutaneous splanchnic nerve radiofrequency ablation for chronic abdominal pain. *ANZ J Surg*. 2005).

The sympathetic innervation of abdominal organs originates from the anterolateral horn of the spinal cord. Preganglionic fibers from the Th5 to Th12 thoracic segments exit the spinal column and merge with the ramus ventralis. These fibers, along with the communicating rami, travel along the truncus sympathicus or sympathetic chain. They do not form synapses within the sympathetic chain but instead traverse it. Synaptic connections form in more peripheral locations, at the level of the ganglion celiacum and ganglion mesentericum superius. These preganglionic nerves converge to form three nervi splanchnici – major, minor, and imus – that course along the paravertebral borders (Table 2). Converging just below the diaphragmatic crus, the splanchnic nerves connect with vagal preganglionic parasympathetic fibers and sensory fibers of the nervus phrenicus, forming the celiac plexus that envelops the abdominal aorta's anterior side. This anatomical arrangement permits precise denervation due to the relatively narrow compartment in which the splanchnic nerves are situated (Figures 1 and 2).

**TABLE 2:** Nervi splanchnici and preganglionic fiber levels.

Nervus splanchnicus division	Preganglionic fiber level
Nervus splanchnicus major	Th5–Th9
Nervus splanchnicus minor	Th10–Th11
Nervus splanchnicus imus	Th11–Th12

###### Interventional pain treatment

Celiac plexus block and splanchnic nerve block can be performed using guidance from various imaging modalities such as fluoroscopy, CT, or endoscopic ultrasound (EUS). Injecting the neural target(s) is possible either unilaterally or bilaterally from an anterior or posterior approach.

Efficacy studies have demonstrated positive outcomes for patients with both pancreatic cancer‐related pain and pancreatitis‐related pain.^104^, ^105^, ^106^ Local anesthetic nerve blocks are often used for prognostic purposes before pursuing neurolytic treatments, which typically prolong the therapeutic effect but carry risks. Utilizing radiofrequency (RF) thermal lesioning of the splanchnic nerves instead of directly treating the celiac plexus with phenol or alcohol has the advantage of preventing or reducing the rate of severe side effects such as retroperitoneal fibrosis, irreversible nerve injury, and potential paraplegia (anterior spinal artery spasm); however, the very small lesions effected by RF neurotomy and the anatomical variability in locations result in a much higher rate of failed nerve capture.^107^ Consequently, recurrent RF denervation of the splanchnic nerves appears to be a safer therapeutic option for patients with non‐malignant pancreatic pain.^3^, ^29^, ^105^ Following a successful chemical or thermal neurolytic block, pain often returns within weeks to months. The effectiveness of a second neurolytic procedure appears to be diminished, at least for celiac plexus neurolysis performed for pancreatic cancer, in part due to the reorganization of damaged nerve connections and disease progression.^108^ Occasionally, a long‐term effect is observed, even with local anesthetic blocks, suggesting potential treatment effects such as recalibrating nerve sensitivities or permanently interrupting the pain cycle (i.e., reversing the processes of central sensitization). Causes for block ineffectiveness include technical failure, aberrant nerve pathways, and possibly psychological factors influencing pain perception.^104^

###### Celiac plexus block

Celiac plexus block has been employed as a pain management technique for pancreatic pain since its first description by Kappis in 1914.^109^ Although not technically demanding, targeting the widespread anatomical location of the celiac plexus can be challenging.^29^ Most evidence is derived from upper abdominal cancer pain studies. Multiple reports have demonstrated significant pain reduction after celiac plexus block in up to 90% of cancer patients, but mainly with limited follow‐up of weeks to months and the continued need for pain medication.^110^, ^111^, ^112^, ^113^, ^114^ Traditionally, the block is performed percutaneously under fluoroscopy or CT imaging, but over the past few decades, EUS is increasingly applied.^90^, ^115^

####### Diagnostic value

Celiac plexus block, ideally with a placebo injection or somatic block (e.g., intercostal nerve block or transversus abdominus plane block) as a control, is used to identify visceral pain and distinguish it from abdominal wall pain. However, there are no high‐quality studies evaluating its validity, and practical concerns (e.g., risks, ethics and cost considerations pertaining to control injections) limit its utility in this setting.^116^

####### Prognostic value

Many practitioners recommend performing a prognostic block before carrying on with a neurolytic celiac plexus block, although evidence for this is limited. Given the potential complications of neurolysis, a diagnostic block with local anesthetic may be useful if the risk of performing two procedures is low. However, if there are high risks or patient burden associated with a second procedure, it may be better to go directly to a neurolytic block. More research is needed to determine the benefits of performing a prognostic block before chemical neurolysis of the celiac plexus (or splanchnic nerves).^117^

####### Clinical effectiveness

Studies in chronic pancreatitis patients are scarce, with no published placebo or sham‐controlled randomized trials.

A recent retrospective study evaluated 62 chronic pancreatitis patients referred to the pain service at their university hospital between 2018 and 2020. In 11 patients, a CT‐guided percutaneous celiac plexus block with local anesthetic and corticosteroid was performed. Seven of them experienced >50% pain relief from baseline. There was a significant decrease in mean NRS score from 9.2 (±0.9) to 4.4 (±3.1) after celiac plexus block. Pain relief lasted on average 69 days (range 0–240 days).^70^

In a single‐center retrospective study evaluating 72 CT‐guided celiac plexus blocks performed for chronic non‐cancer‐related abdominal pain, a subgroup of 3 chronic pancreatitis patients had an effective neurolytic block with phenol lasting an average of 160 (14–390) days. In several patients, a block with local anesthetic alone was performed, which was effective in 9 of 11 patients, with pain reduction for a median of 35 (range 2–135) days.^104^

A retrospective analysis of repeated EUS‐guided celiac plexus block with local anesthetic and steroid over a 17‐year period reported pain relief after the first treatment in 76% of 248 chronic pancreatitis patients with a median duration of 10 (range 1–54) weeks. No major adverse events were reported, suggesting that repeat procedures appear to be safe.^118^

In an older prospective study involving 90 patients who underwent EUS‐celiac plexus block with bupivacaine and triamcinolone for chronic pancreatitis‐related pain, a significant improvement in overall pain scores was reported in 55% of patients at 4‐ and 8‐week follow‐ups. Persistent benefit beyond 12 and 24 weeks was seen in 26% and 10% of patients, respectively, with younger patients (<45 years of age) and those having previous pancreatic surgery being less likely to respond.^119^

A systematic review and meta‐analysis evaluating 221 chronic pancreatitis patients from 6 studies demonstrated that EUS‐guided celiac plexus block (using a local anesthetic and steroid) was effective for treating pain in approximately 50% of patients over a 4–8‐week follow‐up period.^120^ Another review described similar outcomes for EUS‐guided celiac plexus block in 9 studies that included 376 patients with chronic pancreatitis pain, demonstrating pain relief in nearly 60% with follow‐up periods ranging from 1 to 15 weeks.^121^ In most of the included studies, local anesthetic mixed with steroid was used to perform the nerve block, with one exception where a neurolytic block with alcohol was used. Both reviews also evaluated the effect of celiac plexus block in pancreatic cancer pain (in 119 and 283 patients, respectively). They found better results for celiac plexus block in the pancreatic cancer group, with 70%–80% effectiveness. Of note, nearly all patients with pancreatic cancer pain received a neurolytic block with alcohol or phenol, in contrast to the chronic pancreatitis group who were treated with local anesthetic and steroid alone.

EUS and CT‐guided techniques theoretically have several advantages over fluoroscopic guidance. For EUS, these include real‐time color Doppler visualization, improved monitoring of injection into the celiac space and ganglia, and more precise measurements of the anatomical distance to the pleura and spinal nerves. With CT guidance, nervous and visceral structures, scar tissue formation, and altered anatomical relationships after pancreatic surgery are more easily identified.^101^, ^104^, ^105^ However, with EUS‐guided procedures, complication rates between 7% (for local anesthetic nerve blocks) and 21% (for chemical neurolytic blocks) have been reported.^80^ These are mostly minor and transient in nature, including diarrhea (up to 23%), postural hypotension (up to 33%), and pain exacerbation (up to 36%), but major adverse events such as infectious complications and even paraplegia have been occasionally described in the literature.^106^ With fluoroscopy, the major advantage is the very high sensitivity of detecting intravascular spread using real‐time or digital subtraction angiography.

A comparative‐effectiveness study comparing fluoroscopically guided versus an EUS‐guided approach in 56 patients showed a statistically significant improvement in pain scores in 70% of subjects in the EUS group versus 30% in the fluoroscopically guided group. Both groups received local anesthetic with corticosteroid.^122^ In another randomized evaluation performed in 22 patients, EUS‐celiac plexus block was also found to be more effective than a CT‐guided approach at providing analgesic relief (50% vs. 25% with meaningful relief), with 40% experiencing persistent benefit lasting at least 8 weeks.^123^ In a meta‐analysis of the pooled data in these 2 studies, the authors found that EUS‐celiac plexus block was more effective in reducing pain compared to CT‐ or fluoroscopically guided blocks only at 4 weeks, with no statistically significant differences observed at earlier or later time periods.^124^

A randomized double‐blind trial conducted in 40 chronic pancreatitis patients compared local anesthetic alone to steroid plus local anesthetic. The authors reported that only 15% experienced a decrease in the pain disability index at 1 month, with no difference between groups.^125^ Outcomes of this study raise questions as to whether this procedure is an appropriate treatment option for chronic pancreatitis patients altogether.^7^

It is important to note that there are several different ways to perform celiac plexus block including an antero‐crural, transaortic, and a bilateral retrocrural approach that is similar to splanchnic nerve blocks, as well as a transdiscal, retrocrural approach that removes the necessity of performing 2 blocks and can reduce the risk of inadvertent vascular or renal injury. However, entering an intervertebral disc with a needle may be associated with accelerated disc degeneration. Several studies have compared outcomes for different techniques in cancer‐related pain, with most studies being underpowered but demonstrating non‐inferiority between approaches. Being able to vary the approach to celiac plexus block based on anatomical considerations may be more relevant for cancer‐related pain than chronic pancreatitis, with tumor burden potentially limiting the spread of injectate in some patients (e.g., antero‐crural) techniques.^126^, ^127^, ^128^

Currently, most centers do not routinely perform celiac plexus block for chronic pancreatitis pain given the limited evidence for efficacy and short‐lived responses. Additionally, there is a lack of chronic pancreatitis‐specific controlled studies, with much of the evidence being extrapolated from pancreatic cancer pain. Recent international guidelines conclude that celiac plexus block is potentially beneficial in selected cases of painful chronic pancreatitis, but also advise exercising restraint in using it as a routine therapy.^41^, ^54^

For an overview of the literature concerning celiac plexus block in the context of pancreatic cancer pain as well as the technical aspects of the procedure, we refer readers to the publication “Pain in patients with cancer” in the same series as the current review.

###### Splanchnic nerve block and radiofrequency treatment

Many interventional pain physicians specializing in the treatment of abdominal pain have abandoned the classic celiac plexus block in favor of bilateral splanchnic nerve block at the T10–T12 levels. Although some experts have expressed caution, mainly regarding the fear of pneumothorax, mixed evidence indicates that in some patients, this technique may be more effective, possibly with a longer duration of pain relief compared to celiac plexus block.^18^, ^128^, ^129^, ^130^

Retrospective data on 16 patients with chronic visceral abdominal pain including chronic pancreatitis, who underwent both fluoroscopically guided bilateral splanchnic nerve block at the T11 level as well as celiac plexus block with local anesthetic at various time intervals, were analyzed in a recent case series.^129^ Superior improvement in pain scores (reduction of 4.9 points versus 3.1 scored on a visual analogue scale (VAS)), block duration (median of 56 versus 21 days), and patient satisfaction were observed in favor of the splanchnic nerve block.^129^

In patients with upper abdominal malignancy, it has been suggested that greater and sustained pain relief is achieved because the splanchnic nerves may be more preserved and accessible (i.e., less tumor burden hindering the spread of the injectate to the neural target) than those in the preaortic area as the malignancy progresses.^18^, ^130^, ^131^ The same might be true for advanced chronic pancreatitis in the presence of extensive anatomical changes. Moreover, neural blockade at the celiac plexus is not limited to sympathetic fibers or visceral nociceptive afferents but may involve all types of nerves within the ganglia, with widespread anatomical distribution.^129^ In contrast, the narrow anatomical window in which the splanchnic nerves are located allows for more targeted denervation.^3^

The advantage of radiofrequency (RF) treatment is that denervation is performed in a controlled fashion with the lesion limited to the tip of the electrode, thus preventing unwanted spread of neurolytic agents with possible damage to important vascular structures or surrounding tissues, such as nerve roots and the epidural or subarachnoid space.^132^ On the flip side, the small, controlled lesion may be associated with a higher risk of technical treatment failure with fluoroscopically guided procedures (i.e., missed nerves), especially without a reliable method (e.g., sensory stimulation of somatic nerves, motor stimulation) to identify the neural targets.

No placebo‐ or sham‐controlled studies have been performed evaluating RF denervation of the splanchnic nerves. However, case series have reported substantial pain relief, reduced analgesic consumption, and improved quality of life in patients with pancreatic cancer^133^ as well as chronic pancreatitis.^132^, ^134^, ^135^

One of the earliest reports on this topic, primarily covering the technical aspects of the procedure, provided prospective follow‐up data on 69 patients suffering from abdominal pain of various origins, including chronic pancreatitis.^132^ Patients were treated with RF splanchnic nerve lesioning (*n* = 31) or splanchnic nerve block with local anesthetics alone (*n* = 38). All patients had positive prior prognostic blocks before RF lesioning, and treatment was performed bilaterally or unilaterally at the T11 and/or T12 levels. Good to excellent results (≥50% improvement in VAS score) were obtained in 55%–70% of patients, with reduced opioid requirements and improved function, though the latter benefits were not quantified. No clear follow‐up data were reported, but 40% of patients required repeat splanchnic RF at approximately 3 months because of recurrent intolerable pain.

In a retrospective observational study, 10 chronic abdominal pain patients, most with a diagnosis of chronic pancreatitis, underwent percutaneous RF to the splanchnic nerves at the T12 level. Patients reported a significant reduction in mean VAS pain scores, opioid use, acute admissions for pain, anxiety levels, daily activities, overall mood, and general perception of health over a median of 18 (12–24) months follow‐up.^134^

Similarly, another retrospective study evaluated the outcomes of 18 splanchnic nerve RF procedures in 11 patients with refractory chronic pancreatitis pain during a mean follow‐up period of 19 months.^135^ After a positive prognostic block, RF treatment was performed at the T11 and T12 levels unilaterally or on both sides in cases of bilateral pain. The authors found that 78% of patients reported at least a 50% reduction in NRS scores (overall decrease from 7.7 to 2.8), in addition to a reduced need for analgesics. Repeated procedures were performed in seven patients, with approximately 1 year between treatments, resulting in similar pain reduction compared to the primary treatment. In responders – all except three patients – alleviation of pain persisted for a median period of 45 weeks. Only one minor side effect, temporal hypoesthesia of the flank, was reported. The authors emphasized that RF treatment can be successfully repeated after the effect of the previous procedure has subsided as a particular strength compared to other denervation techniques.

A more recent randomized controlled trial conducted in 60 patients with abdominal cancer pain reported better outcomes with fluoroscopy‐guided bilateral RF treatment at the T10–T11 level than with chemical neurolysis of the splanchnic nerves, with more pronounced reductions in pain score (86 versus 43%) and more persistent effects at the end of 3‐month follow‐up compared to the chemical neurolysis group, in which pain returned to baseline levels more rapidly.^136^

The classic posterior approach is the most commonly used method to perform splanchnic nerve block; however, an anterior CT‐guided approach^137^ and a posterior transdiscal approach^138^ are also described in the literature. In addition, thoracoscopic splanchnicectomy has gained interest as a pain treatment for selected chronic pancreatitis patients after the first report in 1994.^139^ Although thoracoscopic splanchnicectomy has the benefit of direct visual guidance and provides pain relief in a substantial number of patients, concerns have been raised about the invasiveness of the procedure,^140^ serious complications such as pneumothorax or intercostal neuralgia,^141^ the development of pleural adhesions,^135^, ^142^ and limited sustained effects.^143^, ^144^ Similar to other surgical procedures, baseline opioid use, disease burden and duration, and pain levels may adversely affect long‐term outcomes,^54^, ^144^ which have not been evaluated in sham‐controlled studies.

International guidelines assert that there is insufficient evidence to recommend either chemical, RF, or thoracoscopic splanchnic nerve block as standard therapy, but all may be considered as a treatment for chronic pancreatitis pain in well‐selected patients when conventional pain therapy is not effective.^41^, ^54^

###### Spinal cord stimulation

Several studies have examined the effectiveness of spinal cord stimulation (SCS) in chronic pancreatitis pain management, although the available evidence is limited and consists primarily of case reports, case series, and retrospective studies. A recent systematic review^140^ included 31 patients from one observational cohort study,^145^ two case series,^146^, ^147^ and four case reports.^148^, ^149^, ^150^, ^151^ These studies generally indicated that SCS can lead to significant reductions in pain intensity, with an estimated 61% (range 50%–100%) median reduction in pain scores at 1‐to‐2‐year follow‐up. Additionally, SCS has been associated with decreased opioid use and reduced pain‐related disability, suggesting its potential to improve the overall quality of life in individuals with chronic pancreatitis. A trial period before permanent implantation was performed in all patients.^152^, ^153^, ^154^ Significant reductions in pain scores and improved quality of life were also seen with off‐label dorsal root ganglion (DRG) stimulation at the T7–T10 levels in a recent case report^13^ and a case series in patients with painful chronic pancreatitis refractory to conventional treatments.^155^

Publications on SCS treatment for chronic abdominal (visceral) pain from varying underlying causes often include patients with painful chronic pancreatitis. Overall, these studies reported decreased pain scores, decreased pain‐related disability, reductions in analgesic consumption, and improvements in quality of life, daily activities, and mood.^149^, ^152^, ^153^, ^156^, ^157^, ^158^, ^159^, ^160^, ^161^ A propensity‐matched analysis of chronic abdominal pain patients that included chronic pancreatitis sufferers receiving either repeated RF ablation of splanchnic nerves or SCS suggested similar outcomes at 3 and 6 months, but at 12 months, improvements in pain scores and opioid usage were significantly less in the SCS group.^162^ The fact that SCS is a (relatively) reversible and minimally invasive therapy is an additional benefit when comparing it to surgical treatment.

Current guidelines do not provide clear recommendations for the timing and circumstances in which SCS should be considered.^54^ Furthermore, reimbursement can be a limiting factor. Patient selection plays a crucial role, with co‐morbid psychological factors, the presence of unexplained abdominal pain suggestive of nociplastic etiology, and opioid dependence adversely affecting outcomes. The success of a positive sympathetic nerve block prior to an implantation trial might also be of predictive value.^140^, ^149^, ^157^

Although promising, more robust research is needed, including randomized controlled trials, to better understand the role and efficacy of SCS in managing chronic pancreatitis pain. Recently, the 4‐month results from the ongoing randomized controlled PANACEA trial – evaluating the effectiveness of SCS in individuals with refractory visceral pain secondary to chronic pancreatitis – were presented. Significant improvements in pain scores, quality of life, and sleep were observed compared to baseline and conservative medical management.^163^

#### Complications of interventional pain management

##### Complications of celiac plexus block

Typical adverse events after celiac plexus block are transient worsening of pain, diarrhea, and hypotension reported in about 40% of patients.^54^ Other described risks are primarily associated with chemical neurolytic blocks and include spinal cord and nerve damage, retroperitoneal and visceral hematoma, abscess, and discitis when a transdiscal approach is used, which may occur despite technical expertise and image‐guided modalities.^105^, ^164^ For spinal cord injury, which can occur with local anesthetic or neurolytic procedures, the deemed mechanism is spasm or damage to the lumbar segmental arteries perfusing the spinal cord (anterior spinal artery syndrome), either by needle trauma or chemical injury.

##### Complications of (radiofrequency) splanchnic nerve block

Pneumothorax, diarrhea, and radiofrequency neuritis can occur following local anesthetic or chemical neurolytic splanchnic nerve blocks or RF lesioning.^3^ Other recorded complications are cardiac arrhythmias induced by phenol in splanchnic nerve blocks and diaphragmatic paralysis.^134^ Although the chance for severe complications is rare when proper technique is used, one should always be cognizant of these. Because of the risk of bilateral pneumothorax, some providers elect to perform bilateral procedures in separate sessions.^135^ In the study by Amr et al.^136^ there were no major complications, but transient paresthesia – caused by alcohol spreading towards thoracic sensory nerves that pass paravertebrally near the splanchnic ganglions – was observed in 70% of neurolysis patients versus none in the RF group. In contrast, more patients in the RF group experienced temporary abdominal colic (73% versus 30%). Diarrhea, hypotension, injection pain, and backache were observed in both groups with no significant difference between groups.

##### Complications of spinal cord and DRG stimulation

In the systematic review by Ratnayake et al.^140^ reported complications secondary to SCS were infrequent, with a 6% infection rate at the lead insertion site and a 6% lead migration rate. However, complication rates after SCS utilized for various other etiologies of chronic visceral pain have ranged from 25%–40%, with lead migration being the most common.^140^, ^158^, ^165^ The most commonly reported procedural or device complications after DRG stimulation are pain at the IPG pocket site, lead fracture, lead migration, and infection.^166^

#### Evidence for interventional management

Table 3 provides a summary of the evidence for interventional pain management techniques for chronic pancreatitis according to systematic reviews.

**TABLE 3:** Summary of the evidence according to the systematic reviews.

Author ‐ date	Technique	Quality of evidence	Conclusion	Recommendation
Verhaegh 2013^135^ Garcea 2004^134^ Raj 2002^132^	Radiofrequency (RF) splanchnic nerve block	*Very low* Case series including 31, 10 and 11 patients, respectively, with chronic abdominal^132^, ^134^ or chronic pancreatitis pain^135^ treated with RF splanchnic lesioning at the T11 and/or T12 level(s)	Results showed substantial pain relief, reduced analgesic consumption, and improved quality of life during 11–18‐month follow‐up	There is very low‐quality evidence that RF treatment of the nervus splanchnicus reduces pain in patients with chronic pancreatitis
Ratnayake 2020^140^	Spinal cord stimulation (SCS)	*Very low* Systematic review including 31 chronic pancreatitis patients from one observational cohort study,^145^ two case series^146^, ^147^ and four case reports^148^, ^149^, ^150^, ^151^	These studies generally indicate that SCS can lead to significant reductions in pain intensity, with an estimated median reduction of 61% (range 50%–100%) in VAS pain scores and 69% (range 25%–100%) median reduction in opioid use at 1‐to‐2‐year follow‐up	There is very low‐quality evidence that SCS has a potential role in reducing pain and opioid use in chronic pancreatitis patients
De Moura 2015^124^	Celiac plexus block (CPB)	*Low* Systematic review and meta‐analysis including 74 chronic pancreatitis patients from 2 RCTs^122^, ^123^ comparing endoscopic ultrasound‐guided (EUS) CPB with CT and fluoroscopically guided CPB	EUS‐CPB was more effective in reducing pain compared to CT or fluoroscopic guidance only at 4‐week follow‐up, with no statistically significant benefit 1, 8 and 12 weeks after the procedure	There is low‐quality evidence that CPB with local anesthetic and corticosteroid reduces pain for a limited time period. The number of responders is higher when treated with EUS‐CPB

### Recommendations

Considering the limited quality of evidence, overall short duration of effect, and invasiveness of the procedure, percutaneous celiac plexus block is not recommended as a pain treatment for chronic pancreatitis pain. [*Strength of recommendation: weak against. Level of evidence: low*].

Based on restricted evidence, radiofrequency of the splanchnic nerves should be considered in patients with chronic pancreatitis pain that is refractory to conventional management. The use of spinal cord stimulation should be limited to research applications and refractory cases associated with major quality‐of‐life decrements, only after the risks and benefits are clearly explained to the patient. [*Strength of recommendation: very weak. Level of evidence: very low*].

#### Clinical practice algorithm

**FIGURE 3:**
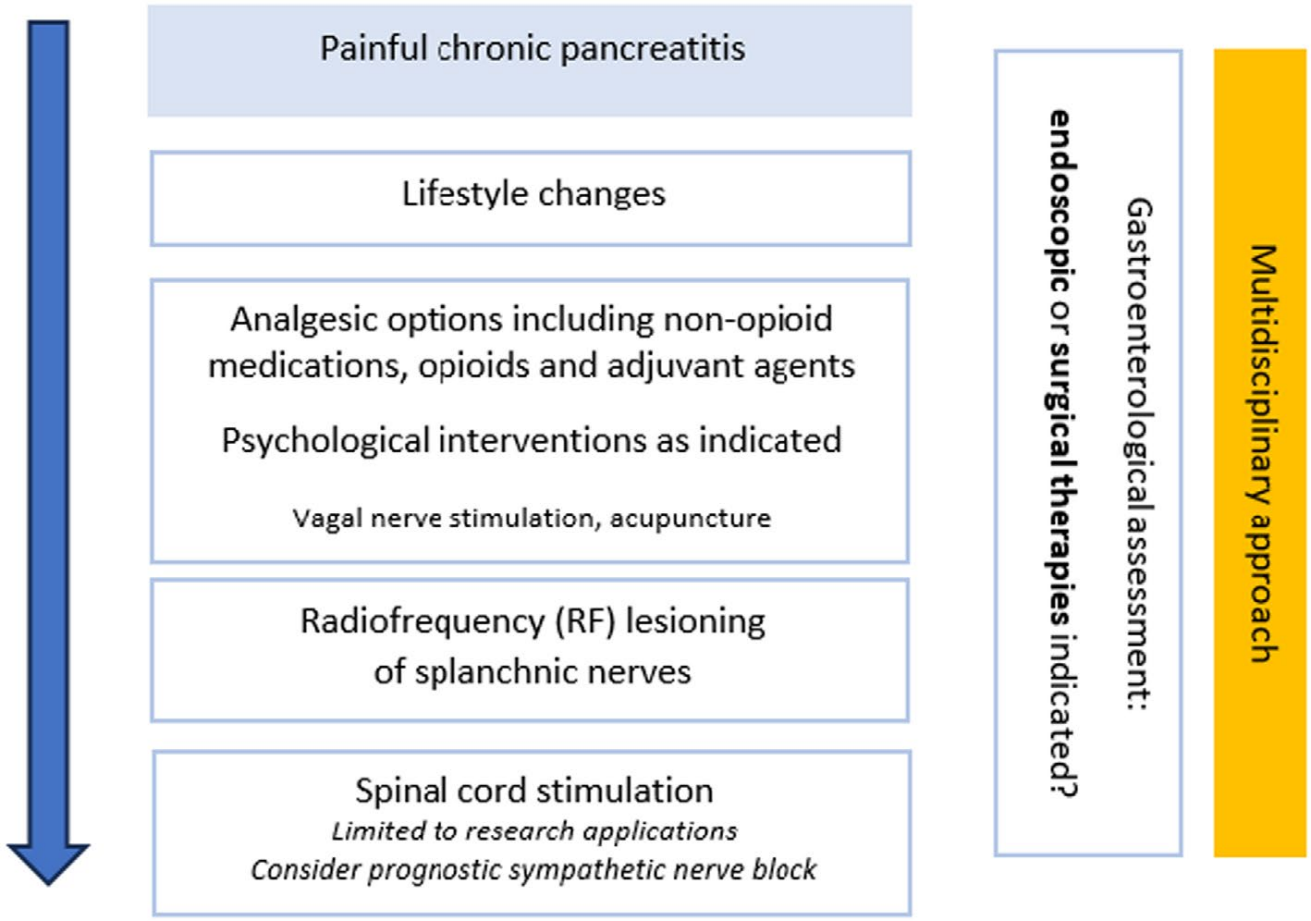
Treatment algorithm for painful chronic pancreatitis.

The algorithm for the interventional pain management of chronic pancreatitis is represented in Figure 3.

#### Technique(s)

Depending on the interventionalist's experience and training, different procedures may be considered when applying interventional pain management strategies. For alternative techniques, we refer readers to “Interventional Pain. A step‐by‐step guide for the FIPP Exam.”^167^ Ultimately, physicians should use techniques they feel most comfortable with.

##### Percutaneous splanchnic nerve block/RF ablation

Generally, a prognostic nerve block can be considered using a similar technique as that described below, ideally with low volumes (<1.5 mL) to enhance specificity and simulate RF lesioning dimensions.^117^

Both splanchnic nerve blocks and RFA are performed under fluoroscopic guidance. The patient is placed in the prone position on a fluoroscopy table. Prior to the procedure, an intravenous infusion line, 3 L/min O_2_ via nasal cannula, an ECG, and saturation monitoring can be installed as indicated. To reduce risks, if sedation is used, the patient should remain responsive to verbal stimuli and breathing spontaneously. Because this is a sympathetic block, hypotension can occur during or after the procedure. This can be treated with intravenous fluid administration.^168^ The T12 and L1 vertebral bodies are identified in a posteroanterior position, and the vertebral endplates are aligned through caudal angulation of the image intensifier. A caudad angle can be used to move up the ribs and transverse processes to facilitate needle placement. The C‐arm is rotated 5–10 degrees to the side to be treated. The patient is asked to breathe in and out deeply. The site of attachment of the diaphragm is identified at mid‐Th12 level. The needle placement site is located where the rib is attached to vertebrae Th11 and Th12, and above the diaphragm. The entry site is marked with a pen. The skin and the deeper layers are infiltrated with local anesthetic (e.g., lidocaine 2%). The RFA treatment is performed with a 10‐ to 15‐cm 20 or 18‐G blunt or sharp curved needle with an active tip of 10 mm, while prognostic blocks should be performed with a smaller gauge needle. As these patients are often very lean, a 10‐cm needle is usually of sufficient length.

**FIGURE 4:**
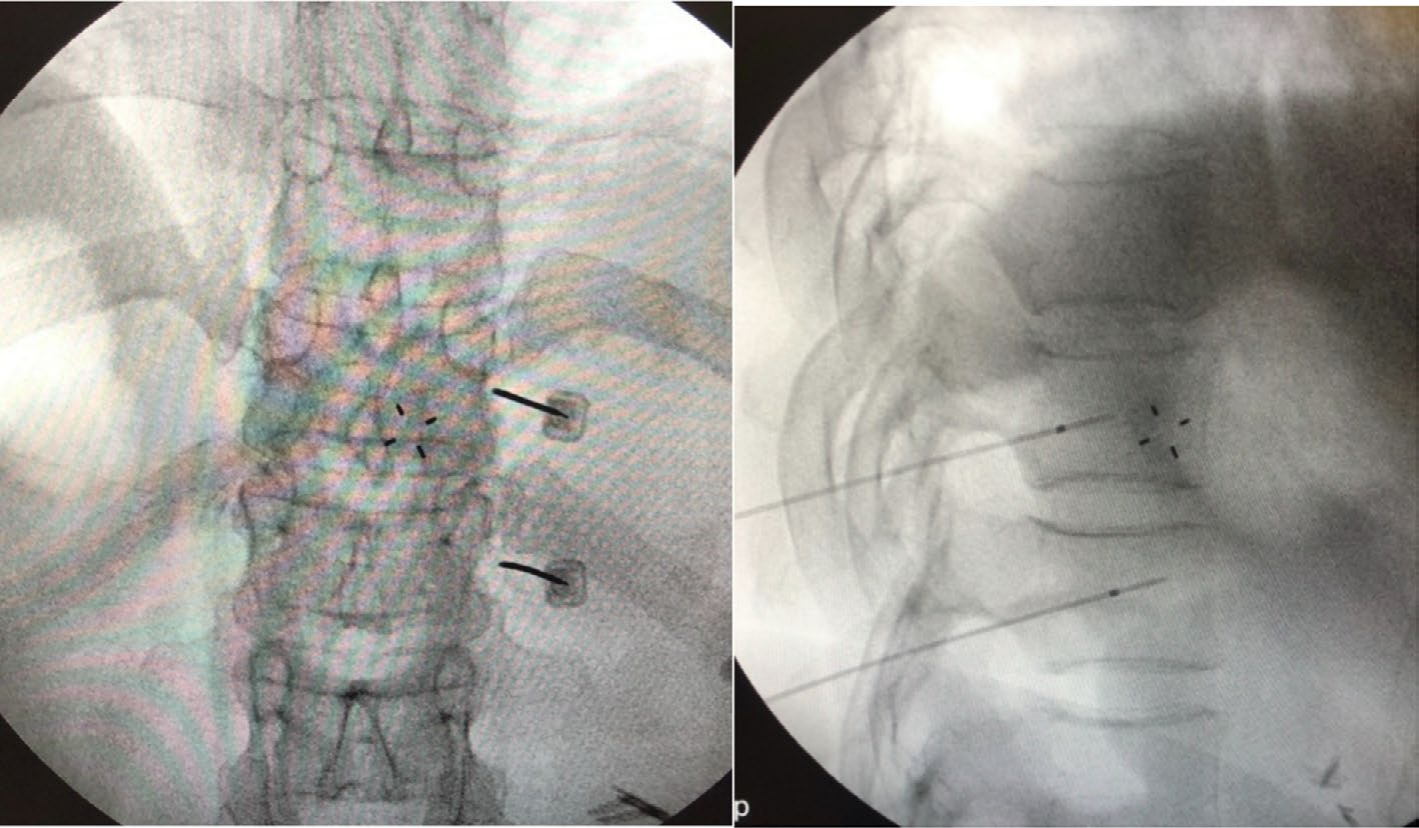
Radiofrequency nervus splanchnicus block at Th11 and Th12 level in anterior posterior and lateral view.

**FIGURE 5:**
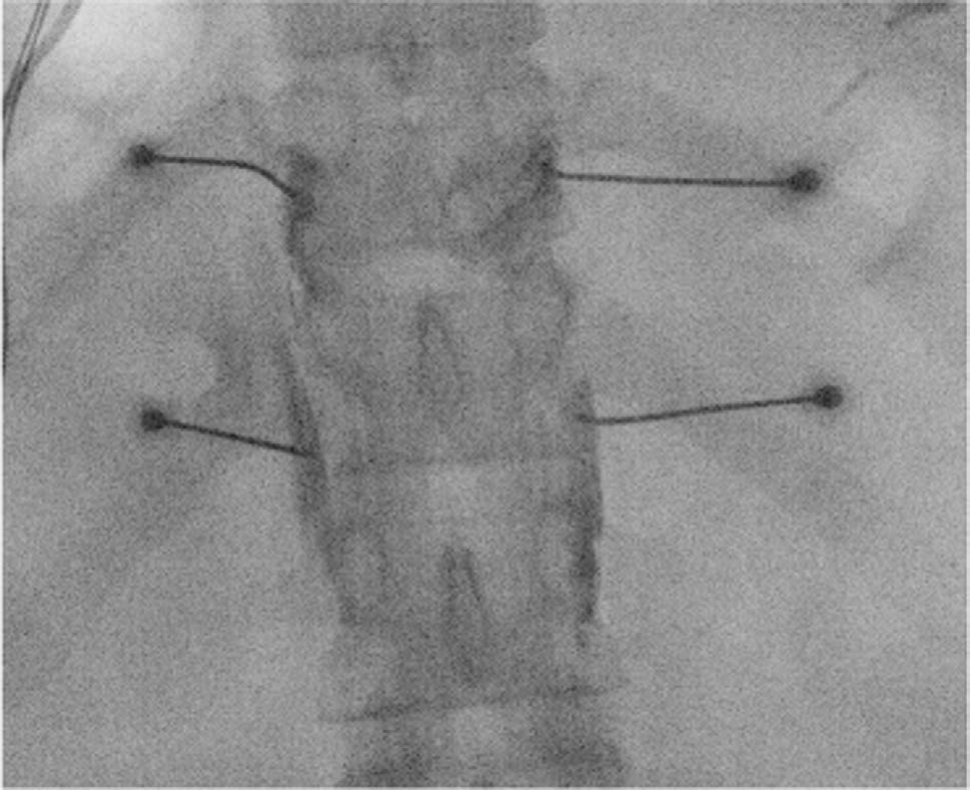
Bilateral needle placement: Contrast spread in anterior posterior view.

When the needle is inserted through skin and subcutis and is in the right direction, the depth is verified in lateral view. The needle must remain posterior to the intervertebral foramen. The C‐arm is returned to a co‐axial view. Initially, the bent tip of the needle is turned outwards to prevent needle passage into the intervertebral foramen. Once the needle has passed beyond the foramen, it is turned so the curve faces medially to maintain close contact with the corpus vertebrae. The needle depth is regularly monitored (every 0.5 cm) using lateral fluoroscopic views. The position of the needle tip generally lies within 5 mm of the corpus vertebrae at the junction between the anterior and middle one‐third of the vertebra, but final positioning is confirmed via electrical stimulation given the variability in anatomy (Figure 4). Regular checks are required to ensure no blood, cerebrospinal fluid (CSF) or chyle appears from the needle depending on the catheter depth. If any CSF or chyle is noticed, the procedure should be discontinued and rescheduled at a later date. If a screening prognostic block has been performed at a previous visit, then the RF cannula should be positioned at the same location under fluoroscopic guidance using the saved block images as a guide. Once a suitable needle location is verified in multiple views, the correct electrode position is confirmed by sensory and motor stimulation. The RF device is connected to the needle, and a grounding pad is applied to the patient. The impedance should be lower than 250 Ohm. The patient should ideally feel a vibrating epigastric sensation at 50 Hz and a threshold of ≤0.5 V. If the electrical sensation is noted in the intercostal area, the needle should be moved further ventrally. In addition, motor stimulation is applied at 2 Hz, focusing mainly on intercostal stimulation. Normally, no distal muscle contractions are observed. If the test results are positive, one milliliter of nonionic, non‐neurotoxic contrast fluid (iohexol) is injected. The contrast should show an overlapping butterfly‐shaped image between the thoracic vertebrae over the lateral vertebral edge (Figure 5) The contrast fluid will fan out in case of an intrapleural location. A local anesthetic is then administered (about 2 mL) to reduce procedure‐related pain and amplify lesion size. The RF treatment can be carried out after several minutes. For each level, the treatment consists of three 90–120 s cycles at 80°C. The needle is first turned with its curve in cranial direction, then to the neutral position, and finally in a caudal direction to maximize surface area.

When contrast spread is deemed sufficient, additional chemical neurolysis with low volumes of phenol or alcohol (e.g., 1–3 mL) is performed by some interventional pain physicians. There are publications describing chemical neurolysis of the splanchnic nerves in cancer pain using larger volumes^136^, ^168^; however, for chronic pancreatitis, the evidence is lacking, and therefore, this should be subject to further study.

## SUMMARY

Chronic pancreatitis is a progressive inflammatory disease of the pancreas that causes irreversible morphological changes to the parenchyma as well as the pancreatic duct. Pain is the most important symptom that greatly influences quality of life. Treatment requires a multidimensional, interdisciplinary approach, in which lifestyle changes and psychological support are essential. If the patient suffers from pseudocysts or obstruction of the ductus choledochus or the duodenum, these should be treated first. Treatment with pancreatic enzyme supplementation, octreotide, antioxidants, vagal nerve stimulation, or acupuncture can be considered, but results have been inconclusive. The use of analgesic medications in general, and opioids in particular, should be accompanied by objective response metrics and, with opioids, risk mitigation strategies. Radiofrequency treatment of the splanchnic nerves – and spinal cord stimulation, preferably in the context of research investigations – can be considered in patients with pain that is refractory to conservative treatment or when pain persists after surgical or endoscopic treatment.

## AUTHOR CONTRIBUTIONS

Laura van Zeggeren and Raha Boelens Nabbi performed the literature search, reviewed the literature, and wrote the article. Monique Steegers, Hjalmar van Santvoort, André Wolff, Leonardo Kapural, and Steven P. Cohen thoroughly reviewed the article and edited the paper. Jan Willem Kallewaard controlled the paper, provided comments, and had full responsibility.

## FUNDING INFORMATION

The authors have no sources of funding to declare for this manuscript.

## CONFLICT OF INTEREST STATEMENT

Leonardo Kapural is an Editorial Board member of Pain Practice and co‐author of this article. To minimize bias, he was excluded from all editorial decision‐making related to the acceptance of this article for publication.

## Data Availability

Data sharing not applicable to this article as no datasets were generated or analyzed during the current study.
